# Effect of Drop Foot on Spatiotemporal, Kinematic, and Kinetic Parameters during Gait

**DOI:** 10.1155/2017/3595461

**Published:** 2017-04-11

**Authors:** Ida Wiszomirska, Michalina Błażkiewicz, Katarzyna Kaczmarczyk, Grażyna Brzuszkiewicz-Kuźmicka, Andrzej Wit

**Affiliations:** Department of Physiotherapy, Józef Piłsudski University of Physical Education in Warsaw, 34 Marymoncka Str., Warsaw, Poland

## Abstract

*Background.* The complexity of the structure and function of a living body can be affected by disorders and can cause various dysfunctions. *Objective.* The aim of this study was to determine compensatory mechanisms in subjects with drop foot during gait. *Methods.* The study evaluated 10 subjects with drop foot (DF) whose results were compared to a group of 10 healthy controls (C). Spatiotemporal, kinematic, and kinetic parameters during the gait cycle were collected using Vicon system synchronized with Kistler platforms. *Results*. Spatiotemporal, kinematic, and kinetic parameters were significantly different between the analysed groups. In the DF group, the subjects walked almost 47% slower and performed 60% less steps per minute compared to the C group. The main problem in the DF group was insufficient ankle dorsiflexion in the 0–10% of the gait cycle. Mean values in the groups during the first 10% of the gait cycle were as follows: DF (−10.42 ± 5.7°) and C (−2.37 ± 1.47°), which affected the substantial differences in the values of muscle torque: DF (0.2 ± 0.1 Nm/kg) and C (−0.26 ± 0.06 Nm/kg). *Conclusions*. Comparative analysis for joint angles and torques demonstrated that the mechanism of compensation is the most noticeable in the knee joint and less in the hip joint.

## 1. Introduction

In the case of temporary or permanent dysfunctions in the area of the motor system, the human body has an ability to use compensatory mechanisms. Compensation is defined as a process aimed at balancing the deficiencies and adjustment to conditions caused by an illness or injury. The phenomenon of compensation of motor organs in the human body is viewed as an ability to replace (through recovery) the function lost by the damaged organ or taking over this function entirely by another healthy organ [1]. The key role in the process is played by specific control and plasticity of the nervous system [2, 3]. In clinical practice, compensation of motor organ dysfunction is often divided into external compensation (e.g., the use of orthopaedic aids) and internal compensation (e.g., when a subject with a shorter lower limb moves on their toes) [4].

The drop foot syndrome represents a problem that is characterized by varied aetiology. The most frequent causes of this type of disability include, for example, cerebrovascular accident, surgical interventions in the area of the fibular nerve and in the lumbar region of the spinal column, radical syndromes at levels L_2_–L_5_, myopathy, multiple sclerosis, cerebral palsy, amyotrophic lateral sclerosis as a complication after hip joint replacement, injuries caused by accidents, and peripheral nerve damage [5]. The symptoms of drop foot include lower leg muscle atrophy, contracture, inability to stand on the heel, and inability to load the lateral side of the foot [1]. Consequently, subjects use what is termed the steppage gait, that is, during the phase of contact with the ground, the toes start the contact, followed by the lateral ridge of the foot, and finally, the heel. The gait is unsymmetrical, inharmonious, and unsure. Instability has a negative effect on maintaining balance. Therefore, the subjects are forced to use specific orthopaedic aids such as casts, walking sticks, ortheses, or custom shoes.

The aim of this study was to analyse the gait of subjects with drop foot by observing the spatiotemporal parameters, angular values, and torques in the sagittal plane of the ankle, knee, and hip joints. The results presented in this study might represent the background for explanation of the mechanisms of compensation in the people with drop foot based on the comparison with the group of healthy subjects.

## 2. Methods

### 2.1. Participants

Data for this research were collected at a Central Research Laboratory AWF Warsaw. The study evaluated the walking of subjects with drop foot condition (DF) as compared to a gait of healthy subjects (C). The subjects' biodata are summarized in Table 1. The study was conducted according to the ethical guidelines and principles of the Declaration of Helsinki. All subjects gave their informed written consent to the experimental procedures, which were approved by The Senate Ethics Committee of Scientific Research AWF Warsaw.

The group of subjects with drop foot syndrome (DF) suffered from the degenerative disc disorder at the level of L_4_/L_5_ and/or L_5_/S_1_, weakness of ankle dorsiflexion, with particular focus on the tibialis anterior muscle, atrophy or substantial weakening of fibularis muscles, and lower leg and foot numbness. The group of healthy subjects (C) consisted of 10 students. All results obtained in the C group were used as reference values for achievement of the study aim.

### 2.2. Instrumentation and Data Collection

The motion capture system (Vicon Motion Systems Ltd, Oxford, UK) consisted of a set of 9 cameras with infrared detectors was used to record the gait at the sampling rate of 100 Hz. The cameras were set up to record the walking gait over a 10-metre path. Force plates (Kistler Holding AG, Winterthur, Switzerland) were placed along this path to record ground reaction forces of the movements at the frequency of 1000 Hz. First, anthropometric measurements were taken for each person. Next, spherical markers were placed at anatomical landmarks according to the standards of the biomechanical model PlugInGait [6] available within the motion capture system. Each subject performed three walking trials, but analysis was conducted on one trial that does not have recording errors. The recording technique and the software allowed three-dimensional reconstruction of the motion in the major joints of lower extremities. Biomechanical model PlugInGait uses Cardan angles and inverse dynamics tool in order to calculate joint angles and torques [6, 7]. Therefore, for each subject, the spatiotemporal, kinematics (joint angles), and kinetics (joint torques) parameters were recorded. Spatial parameters included stride and step lengths [m], while temporal parameters contained cadence [steps/min], stride time [s], and walking speed [m/s].

### 2.3. Data Reduction

**Figure 1:**
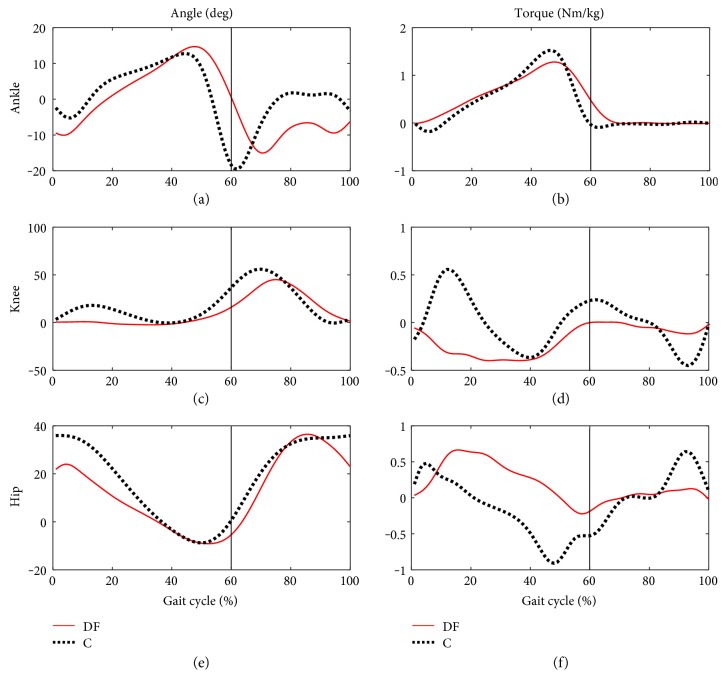
Mean profiles of angles and muscle torques in the gait cycle for the group of people with symptoms of drop foot (DF) and healthy controls (C); (a) angle in the ankle, (c) knee, (e) hip; (b) muscle torque in the ankle, (d) knee, (f) hip.

Figure 1 presents average trajectories of angles and torques in ankle, knee, and hip joints during gait cycle in the DF and C groups, respectively.

In order to compare the curves of angles and torques in joints of the lower limbs during gait between the DF group and the mean values of these variables (${\bar{Z}}_{St}$, ${\bar{Z}}_{Sw}$) in the C group, the procedure of locale extremes was used. The stage in determination of the local extremes (minimums and maximums) for each curve was as follows. Within the stance (St) and swing phases (Sw) of the gait cycle (GC), the values of local extremes *E*^St^ and *E*^Sw^ were determined, assuming that a curve with the values in the ordered set, defined on the topological space, has a local minimum (maximum) in the point *x*_*o*_ of this space if there is an open neighborhood *U* of the point *x*_*o*_ so that for each *x* ∈ *U* *f*(*x*) ≤ (≥)  *f*(*x*_*o*_). Next, the index of difference for the stance (*R*^St^) and swing (*R*^Sw^) phases, for *i* − subject (*i* = 1, 2,…, 10), was calculated using

(1) $$\begin{array}{c} R_{i}^{St}={\bar{Z}}_{St}-E_{i}^{St},\\ R_{i}^{Sw}={\bar{Z}}_{Sw}-E_{i}^{St}. \end{array}$$

In order to compare the indices of differences *R*^St^ and *R*^Sw^ in individual joints, the values of arithmetic means were determined for the profiles of changes in angles and torques for the stance phase ${\bar{R}}^{St}$ and swing phase ${\bar{R}}^{Sw}$:

(2) $$\begin{array}{c} {\bar{R}}^{St}=\frac{\sum_{i=1}^{10}R_{i}^{St}}{10},\\ {\bar{R}}^{Sw}=\frac{\sum_{i=1}^{10}R_{i}^{Sw}}{10}. \end{array}$$

The Shapiro-Wilk test and Mann-Whitney *U* test were used in order to carry out the statistical analysis of the spatiotemporal parameters obtained for the groups C and DF and mean values of the indices of differences ${\bar{R}}^{St}$ and ${\bar{R}}^{Sw}$ between the profiles of angles and torques. Statistical analysis was carried out using Statistica 10.0 software (StatSoft Inc., Tulsa, USA) at the level of significance set at 0.05.

## 3. Results

Gait of subjects with drop foot differed significantly from the gait of healthy controls, which was reflected by the values of basic parameters that describe the gait (Table 2).

In the DF group, the subjects walked almost 47% slower and performed 60% less steps per minute compared to the group C. A direct cause of such results is the more cautious gait with distinct hobbling, typical of the DF group.

Similar to spatiotemporal parameters, the analysis of kinematic and kinetic parameters revealed statistically significant differences between the groups (Table 3).

The major problem of the subjects with drop foot is the small range of the ankle dorsiflexion in the first 10% of gait cycle. Mean values in the groups in this phase were, respectively, DF (−10.42 ± 5.7°) and C (−2.37 ± 1.47°), which causes substantial differences in the values of torques: DF (0.2 ± 0.1 Nm/kg) and C (−0.26 ± 0.06 Nm/kg). Consequently, the compensatory mechanism is observed, causing the greatest differences between the groups in the knee joint in the stance phase both for the values of angles and torques. This is demonstrated by the values of the locale extremes, which were DF (0.91 ± 1.28° and −0.40 ± 0.28 Nm/kg) and C (20.39 ± 1.2° and 0.56 ± 0.21 Nm/kg). This result points to a characteristic function of the knee joint extensors, where the knee bends backwards (hyperextension). This is a characteristic of the DF group. Similar to the knee joint, the profiles of mean angles in the hip joint point to the highest differences in the stance phase: C (36.05 ± 0.9°), DF (21.82 ± 2.3°).

**Figure 2:**
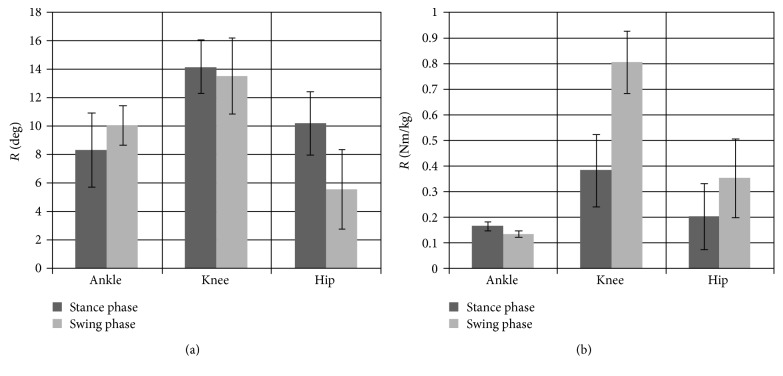
Mean values and standard deviations for the coefficient of differences between extreme points for (a) angle and (b) torque in the stance and swing phases in joints of the lower limb during gait cycle.

Determination of extreme values for changes in angles and torques in joints of the lower limb during the stance phase and the swing phase was used in the key stage of the analysis (Figure 2).

Mean values of ${\bar{R}}^{St}$ and ${\bar{R}}^{Sw}$ calculated from the angles (Figure 2(a)) in the stance phase and the swing phase for each joint of the lower limb point to the significant (*p* < 0.05) initial increase in the difference between the parameters by approximately 41% in the stance phase and 26% in the swing phase between the ankle and knee joints. Furthermore, a statistically significant (*p* < 0.05) reduction in the value of the parameters of differences by 39% in the stance phase and by 57% in the swing phase was observed between the knee and hip joints, with greater differences between the groups DF and C occurring in the stance phase. The same procedures of analysis were carried out for the torques (Figure 2(b)). A significant increase (*p* < 0.05) in the mean value of differences by 44% and 83% in the stance and swing phases, respectively, was found when comparing the ankle and knee joints. We also found that the differences between the knee and hip joints were significantly (*p* < 0.05) reduced by 56% in the stance phase and by 47% in the swing phase. It was also observed that mean differences in torques in individual joints were statistically higher in the swing phase compared to those in the stance phase.

## 4. Discussion

The symptom of drop foot represents a difficult clinical problem, especially in the process of recovery of the normal gait pattern [8]. Measurements of kinetic and kinematic parameters reflect the effect of damage through different spatiotemporal parameters compared to healthy subjects [9]. The values of spatiotemporal, kinematic, and kinetic parameters concerning the gait in people with drop foot symptom and healthy people are consistent with those documented in the other studies [8–10]. Subjects with drop foot walk slower and have to make more and shorter steps in order to move over the same distance as healthy subjects (Table 2). They need more time during the phase of double support, which protects them from uncontrollable fall and helps maintain balance. However, this type of gait involves greater energy expenditure and causes difficulties in everyday life [10].

In the group with drop foot (DF), plantarflexion is dominant in the first 10% of the gait cycle (−10.42 ± 5.7°), whereas in the group of healthy subjects (C) the predominance of dorsiflexion can be observed (−2.37 ± 1.47°). Therefore, in the limb affected by the dysfunction, no phase of the first contact of heel with the ground is observed and the load is taken over by the forefoot. Weakened ankle dorsiflexors and increasing fatigue in the ankle plantarflexors which are contractured with time cause uncontrollable drop and additional load in this joint in the first 10% of the gait cycle [1], which is reflected by greater differences in the values of torques in the groups studied: DF (0.2 ± 0.1 Nm/kg), C (−0.26 ± 0.06 Nm/kg). This has a direct effect on stabilization of distal insertion of the triceps surae muscle group which initiates inhibitory action in order to accelerate the centre of gravity of trunk. Assuming that the line of gravity goes through the knee joint, it is possible for the gastrocnemius muscle to take over active control of stabilization [4]. This mechanism is reflected by the extension (0.91 ± 1.28°) in the knee joint that is maintained by approximately 50% GC. In this case, this is the internal compensatory mechanism which is of degenerative character since it causes premature damage in the knee joint [11], causing secondary overload changes in the patellofemoral joint [4]. Therefore, the subjects with drop foot use the mechanism of the anterior pelvic tilt and trunk tilt in the direction of movement. Consequently, this position of the pelvis causes excessive tension in the muscles of the ischiocrural group, which are biarticular muscles [12]. Coactivation of this group with the gastrocnemius muscle will cause limitation of the flexion at the moment of loading in the stance phase in the hip joint [12]. This is supported by the results of the previous studies [10], where, only after a division of the group of subjects with drop foot into the group characterized by weakened plantar flexors and the group with predominance of drop foot symptoms, the researchers identified compensatory strategies in the hip joints in the swing phase, with increasing angle of the hip joint that prevents irritation of skin of toes against the ground.

The summary and also the principal part of the analysis of kinematic and kinetic parameters are represented by the diagrams of mean values and standard deviations of the coefficient of differences for extreme points between the values of angles and torques (Figure 2) in individual joints in the stance and swing phases. Mean values of parameters ${\bar{R}}^{St}$ and ${\bar{R}}^{Sw}$ calculated for the angles point to the initial increase by approximately 41% between the ankle and knee joints in the phase of stance and by approximately 57% decrease in the difference in the swing phase between the knee and hip joints. Furthermore, analogous analysis of the muscle torques shows that mean difference between the ankle and knee joints increases by 83% during the swing phase. It can be also observed that the differences between the knee and hip joints are substantially reduced by approximately 56% in the stance phase. The study demonstrates that, as a result of dysfunction, the whole kinematic chain is disturbed, which consequently causes that the motor system works under conditions of abnormal load. The seemingly insignificant disturbance in the form of excluding a single muscle group from activity, which in this study means function of dorsiflexors in the ankle joint, causes a substantial reorganization of the proper pattern of the human motor system, controlled by the nervous system and the necessity to utilize the compensatory mechanism. Replacement of the lost function is possible through cooperation of the muscle groups with biarticular muscles performing the eccentric work in the one joint and concentric in the other.

## 5. Conclusions

The investigations presented in this paper demonstrated that inefficiency of one structure in the lower limb not only does affect the gait cycle pattern but also is the cause of pathologies in the regions completely unrelated to the location of the primary disturbance. Using gait analysis techniques allowed us to precisely characterize the gait of subjects with drop foot, which is an important milestone for future research into the management of this disorder and may also have applications in routine clinical care. Therefore, this work may serve as a framework to establish the most appropriate rehabilitative, orthotic, and surgical treatments to prevent ambulatory impairments.

## Figures and Tables

**Table 1:** Characteristics of the groups analysed in the study.

Groups	Age (years)	Height (cm)	Body mass (kg)
	(mean ± SD)	(mean ± SD)	(mean ± SD)
DF (*n* = 10)	45.5 ± 17.4	173.9 ± 12.7	75.7 ± 22.4
C (*n* = 10)	22.6 ± 4.6	174.1 ± 8.6	69.4 ± 11.7

SD; standard deviation; DF: drop foot group; C: control group.

**Table 2:** Values of parameters that describe spatiotemporal structure of gait in studied groups.

Spatiotemporal parameters	DF group (*n* = 10)	C group (*n* = 10)	*p* value
	(mean ± SD)	(mean ± SD)	
Cadence (steps/min)	71.0 ± 9.6	119.4 ± 8.3	0.0002^∗^
Stride time (s)	1.7 ± 0.4	1.0 ± 0.3	0.0417^∗^
Stride length (m)	1.1 ± 0.1	1.5 ± 0.1	0.0001^∗^
Step length (m)	0.5 ± 0.1	0.7 ± 0.1	0.0001^∗^
Walking speed (m/s)	0.7 ± 0.1	1.4 ± 0.1	0.0000^∗^

SD: standard deviation; DF: drop foot group; C: control group; ^∗^significant *p* values (≤0.05).

**Table 3:** Extreme values of angles and torques in lower limb joints during gait of studied groups.

Kinematic and kinetic parameters	Joint	Stance phase		Swing phase	
		min ± SD	max ± SD	min ± SD	max ± SD
*C group*					
Angle (°)	Ankle	−2.37 ± 1.47	12.67 ± 0.7	−19.81 ± 0.9	1.83 ± 1.3
	Knee	0.19 ± 1.28	20.39 ± 1.2	0.07 ± 1.3	55.96 ± 1.2
	Hip	−8.78 ± 1.16	36.05 ± 0.9	0.59 ± 1.10	35.86 ± 1.3

Torque (Nm/kg)	Ankle	−0.26 ± 0.06	1.52 ± 0.78	−0.09 ± 0.78	0.02 ± 0.92
	Knee	−0.37 ± 0.14	0.56 ± 0.21	−0.45 ± 0.45	0.24 ± 0.78
	Hip	−0.93 ± 0.13	0.54 ± 0.34	−0.54 ± 0.65	0.66 ± 1.01

*DF group*					
Angle (°)	Ankle	−10.42 ± 5.7	14.71 ± 2.3	−15.19 ± 3.1	0.52 ± 2.11
	Knee	0.91 ± 1.28	14.33 ± 1.1	1.74 ± 1.3	40.18 ± 1.1
	Hip	−9.08 ± 2.46	21.82 ± 2.3	−5.4 ± 2.18	36.51 ± 2.1

Torque (Nm/kg)	Ankle	0.2 ± 0.1	1.28 ± 0.67	−0.01 ± 0.13	0.11 ± 0.1
	Knee	−0.4 ± 0.28	−0.01 ± 0.56	−0.12 ± 0.23	0.01 ± 0.78
	Hip	−0.23 ± 0.25	0.67 ± 0.13	−0.19 ± 0.13	0.13 ± 0.45

SD: standard deviation; DF: drop foot group; C: control group.
